# Comprehensive Proteomic Profiling of Vitreous Humor in Ocular Sarcoidosis Compared with Other Vitreoretinal Diseases

**DOI:** 10.3390/jcm11133606

**Published:** 2022-06-22

**Authors:** Hiroyuki Komatsu, Yoshihiko Usui, Kinya Tsubota, Risa Fujii, Takefumi Yamaguchi, Kazuichi Maruyama, Ryo Wakita, Masaki Asakage, Hiroyuki Shimizu, Naoyuki Yamakawa, Naoya Nezu, Koji Ueda, Hiroshi Goto

**Affiliations:** 1 Department of Ophthalmology, Tokyo Medical University, Tokyo 160-8402, Japan; v06058@gmail.com (H.K.); tsubnkin@hotmail.co.jp (K.T.); ryo.0623.w@gmail.com (R.W.); patty.m.best@gmail.com (M.A.); sardine_harbor@yahoo.co.jp (H.S.); yamakawa@tokyo-med.ac.jp (N.Y.); naoya.nezu@gmail.com (N.N.); goto1115@tokyo-med.ac.jp (H.G.); 2 Project for Realization of Personalized Cancer Medicine, Cancer Precision Medical Center, Japanese Foundation for Cancer Research, Tokyo 135-8550, Japan; risa.fujii@jfcr.or.jp (R.F.); koji.ueda@jfcr.or.jp (K.U.); 3 Department of Ophthalmology, Tokyo Dental College Ichikawa General Hospital, Chiba 272-8513, Japan; tym.i.eye.i@gmail.com; 4 Department of Ophthalmology, Osaka University Graduate School of Medicine, Osaka 565-0871, Japan; kazuichi.maruyama@gmail.com

**Keywords:** proteomics analysis, ocular sarcoidosis, uveitis, vitreoretinal diseases, vitreoretinal lymphoma

## Abstract

Ocular sarcoidosis is an inflammatory disease that manifests as uveitis, and is often difficult to distinguish from other forms of uveitis based on nonspecific findings alone. Comprehensive proteomic analyses of vitreous humor using LC-MS/MS were performed in each patient with ocular sarcoidosis, vitreoretinal lymphoma (VRL), and controls with epiretinal membrane or macular hole. Differential expression proteins (DEPs) were identified by comparing with VRL and controls, and functional pathway analysis was performed. The candidate biomarker proteins for ocular sarcoidosis were validated using enzyme-linked immunosorbent assay. A total of 1590 proteins were identified in all samples. Of these, 290 and 174 DEPs were detected in vitreous of ocular sarcoidosis compared with controls and VRL, respectively. Enrichment pathway analysis revealed that pathways related to the immune system were most upregulated. Validation of two candidate biomarkers for ocular sarcoidosis, neutrophil gelatinase-associated lipocalin (NGAL) and junctional adhesion molecules B (JAMB), confirmed upregulated NGAL and JAMB protein expressions in ocular sarcoidosis compared to controls and VRL. The results of this study revealed that altered vitreous protein expression levels may discriminate ocular sarcoidosis from other uveitis diseases. Vitreous NGAL and JAMB are potential biomarkers and may serve as an auxiliary tool for the diagnosis of ocular sarcoidosis.

## 1. Introduction

Uveitis is a sight-threatening intraocular inflammation and the leading cause of irreversible blindness in the developed world [1]. Intraocular inflammation with various etiologies has been classified into different disease entities exhibiting heterogeneous clinical features [2], and many forms of uveitis are sufficiently diagnostic by disease-specific clinical findings. However, atypical and nonspecific manifestations of uveitis confound the diagnosis, presenting challenges for clinicians. Ocular sarcoidosis, one of the uveitis forms, is an inflammation eye disease with unknown etiology. Three epidemiological surveys of uveitis conducted in Japan over the past 15 years have reported that sarcoidosis is the most frequent form of uveitis [3,4,5]. Vitreoretinal lymphoma, on the other hand, is an intraocular malignancy that constitutes a subgroup of primary central nervous system lymphomas, and the above-mentioned surveys have indicated an increase in incidence from 1.1 to 2.6% in recent years [3,4,5]. Although the two diseases have different pathogenesis, both manifest similar nonspecific uveitic findings of vitreous opacity, and distinction between the two entities is often difficult. Meanwhile, these diseases are associated with systemic disorders, and prompt and accurate differential diagnosis is important for initiation of appropriate systemic and local treatments to reduce the risk of vision loss and decline of quality of life. This background prompted our search for disease-specific biomarkers as an aid to diagnosis.

Proteomics is a promising bioinformatics tool aiming at advancing the diagnosis and elucidating the pathogenesis of diseases. Proteomic analysis in the ophthalmology field has been performed on various ocular tissues including aqueous humor, retina, and vitreous humor [6,7,8,9,10,11]. Notably, vitreous humor has numerous proteins that differ from serum protein components [8,12,13]. Vitreous proteomics has been used to study different uveitis etiologies, contributing to the elucidation of the pathogenesis of intraocular inflammation [14,15,16]. We previously conducted proteomic analyses using vitreous humor [16,17]. However, with the recent development of bioinformatics technology that allows high-throughput analysis, more information can be obtained at lower cost which facilitates the identification of novel uveitis biomarkers. In addition, although the association of *Cutibacterium acnes* (*C. acnes*) is suspected to be a cause of ocular sarcoidosis [18], detailed pathophysiology remains to be elucidated. Therefore, comprehensive proteomic profiling using vitreous samples from eyes with sarcoidosis may help elucidate the pathophysiology of this disease. Furthermore, to the best of our knowledge, there is no report of comprehensive vitreous analysis comparing sarcoidosis with VRL. The results of comprehensive proteomic profiling using vitreous samples from eyes with different vitreoretinal diseases in this study provide insights into the pathogenesis of ocular sarcoidosis and suggest biomarkers for ocular sarcoidosis.

## 2. Materials and Methods

### 2.1. Patients

Eighty-nine patients were included in this study, comprising 28 with ocular sarcoidosis, 25 with VRL, seven with Bechet’s disease (BD), and 30 controls with epiretinal membrane or macular hole diagnosed at Tokyo Medical University Hospital in 2017–2019. Patients with ocular sarcoidosis or VRL who had not been treated with corticosteroids within the past one month before vitrectomy were enrolled. Excluded from the study were patients with preoperative trauma, vitreous hemorrhage, pre-existing macular pathologies including age-related macular degeneration, and immunodeficiency. Ocular sarcoidosis, VRL, and BD were diagnosed according to current diagnostic criteria based on clinical characteristics, radiographic examination, blood tests, vitreous cytopathological examination, and molecular genetic (such as gene rearrangement) analyses, as described previously [19,20,21,22].

**Figure 1:**
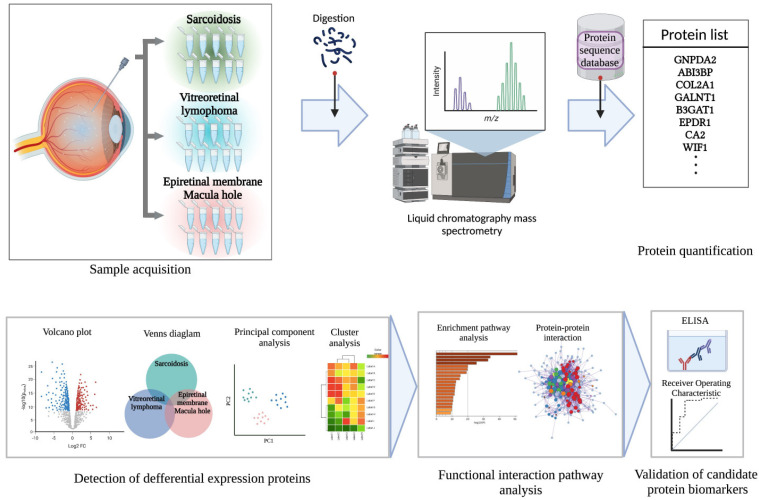
Schematic overview of the present study. Vitreous samples were collected from 10 patients with ocular sarcoidosis, 10 with vitreoretinal lymphoma (VRL), and 10 controls with epiretinal membrane or macular hole. Vitreous samples were pretreated by enzyme digestion, and peptide fragments were detected using liquid chromatography–mass spectrometry. Proteins were identified using a protein sequence database. Next, to detect differential expression proteins among the diseases, various analyses comprising Venn diagram, volcano plot, principal components analysis, and clustering analysis were performed. Functional interaction pathway analyses were conducted to further identify candidate biomarkers. Then, candidate protein biomarkers for ocular sarcoidosis were validated using ELISA to measure protein concentrations in vitreous samples of patients.

First, we performed a comprehensive proteomic analysis of 10 cases each of ocular sarcoidosis, VRL, and controls. Then we validated the results with 27 ocular sarcoidosis cases, 22 VRL cases, 20 control cases, and 7 BD cases (this study population included the cohort in the above-mentioned comprehensive proteomic analysis). The strategy of this study is summarized in Figure 1. The patient’s demographic data for each disease is summarized in Table 1, and detailed clinical data are listed in Table S1. The vitreous samples used in this study were collected when the patients underwent diagnostic or therapeutic vitrectomy [23]. The patients in the control group consisted of 12 with epiretinal membranes and 10 with macular holes, who underwent vitreous surgery for treatment. This study was approved by the Ethics Committee of the Tokyo Medical University Hospital, Tokyo, Japan (SH3281). Written informed consent was provided by all participants in the study. All investigations were conducted in accordance with the principles of the Declaration of Helsinki.

### 2.2. Vitreous Humor Sample Collection and Preprocessing

Vitreous humor samples (approximately 0.5 to 1.0 mL) were collected from the mid-vitreous region at the start of a standard 3-port 25-gauge vitrectomy. The samples were removed with a vitreous cutter before intraocular infusion. The vitreous samples collected were delivered immediately to the Cytology and Molecular Laboratory (ARL, Tokyo, Japan) for tests for the diagnosis of VRL and ocular sarcoidosis. The samples were centrifuged at 3000× *g* for 3 min, and the supernatants were collected and stored at −80 °C until use.

### 2.3. Mass Spectrometric Analysis

Sample processing for proteome analysis was performed according to the previous report [24]. Briefly, vitreous samples were digested with Trypsin/Lys-C Mix (Promega, #V5073). The resulting peptides were analyzed using UltiMate 3000 RSLCnano-flow HPLC (Thermo Fisher Scientific, Waltham, MA, USA) equipped with 0.075 mm × 250 mm AURORA column (IonOpticks, Melbourne, Australia) combined with Orbitrap Fusion Lumos Mass Spectrometer (Thermo Fisher Scientific). From the datasets obtained from LC-MS/MS, the proteins were identified by searching the SwissProt Human Database using the Mascot (Matrix Science, London, UK) or Sequest HT (Thermo Fisher Scientific) search engine. Protein identification and quantification was performed using the Proteome Discover 2.4 software (Thermo Fisher Science).

### 2.4. Bioinformatic Analysis and Statistical Analysis

To detect the changes in protein expression in each disease, the proteins identified by LC-MS/MS were analyzed by various methods including Venn diagram, volcano plot, principal component analysis, and hierarchical clustering analysis. Venn diagrams were analyzed using jVenn (http://jvenn.toulouse.inra.fr/app/index.html, accessed on 18 June 2022) and the figures were generated using BioRender (https://biorender.com/, accessed on 18 June 2022). Volcano plots were analyzed and generated using GraphPad Prism 9. Principal component analyses were performed using MetaboAnalyst 5.0 (https://www.metaboanalyst.ca/, accessed on 18 June 2022). Hierarchical clustering analyses were performed and visualized using Morpheus (https://software.broadinstitute.org/morpheus/, accessed on 18 June 2022). The functional interaction pathways were evaluated by enrichment pathway analysis using Reactome (https://reactome.org/, accessed on 20 June 2022). Protein–protein interaction networks were visualized using Metascape (https://metascape.org/, accessed on 20 June 2022).

### 2.5. Validation of Proteomic Analysis

To quantify the changes in vitreous concentrations of specific candidate biomarker proteins, enzyme-linked immune sorbent assay (ELISA) was performed on vitreous humor samples using Human Lipocalin-2 ELISA Kit (Abcam) and Human JAM-B ELISA Kit (MyBioSource, San Diego, CA, USA), according to the manufacturers’ instructions.

### 2.6. Statistical Analysis

All statistical analyses were performed using SPSS (IBM, Armonk, NY, USA) and R (4.0.0.) (R Foundation for Statistical Computing, Vienna, Austria). A *p* value less than 0.05 was considered significant. Data are expressed as mean ± standard deviation. Comparison of the expressed proteins between diseases was performed using Student’s *t*-test and adjusted using the false discovery rate (FDR). Differential expression protein (DEP) was defined as a protein that satisfied the criterion of FDR-adjusted *p* value less than 0.05. The protein concentrations of each candidate biomarker measured by ELISA among diseases were compared using Kruskal–Wallis test followed by post hoc Mann–Whitney test. Bar plot and receiver operating characteristic (ROC) curve were generated using GraphPad Prism 9 (GraphPad Software, Inc., San Diego, CA, USA).

## 3. Results

### 3.1. Differential Expression Proteins in Vitreous

**Figure 2:**
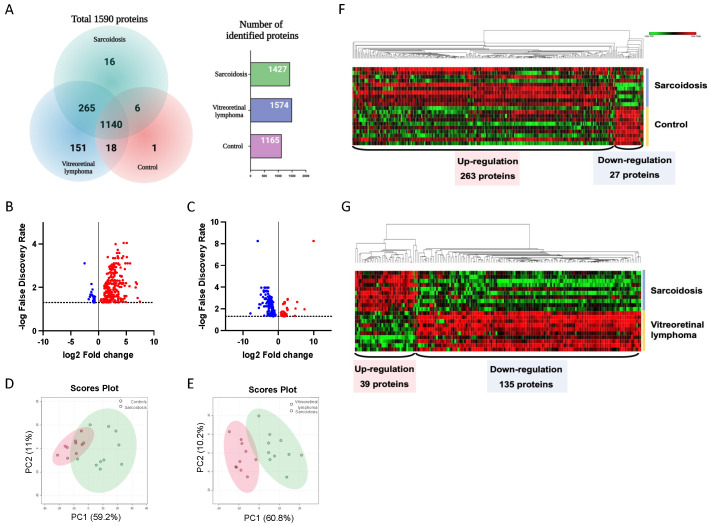
Identification of differential expression proteins. A total of 1590 proteins were detected in this study. Of these, 1140 were common proteins detected in ocular sarcoidosis, vitreoretinal lymphoma (VRL), and controls, whereas 16 proteins were detected only in ocular sarcoidosis and 151 proteins only in VRL (**A**). Differential expression proteins (DEPs) were defined as FDR−adjusted *p* value less than 0.05. The volcano plot showed that 290 and 174 proteins were upregulated (red) or downregulated (blue) compared with controls (**B**) and VRL (**C**), respectively. Principal component analysis visually depicted clear separation of ocular sarcoidosis from controls (**D**) and VRL (**E**). Hierarchical clustering showed that 263 proteins were upregulated while 27 were downregulated in the vitreous in ocular sarcoidosis compared with controls (**F**), whereas 39 proteins were upregulated and 135 were downregulated in ocular sarcoidosis compared with VRL (**G**).

A total of 1590 proteins were identified in all vitreous samples using high-throughput proteomic analysis. Of the 1590 proteins, 1427, 1574 and 1165 vitreous proteins were detected in eyes with ocular sarcoidosis, VRL and control patients, respectively (Table S2). Furthermore, among 1590 proteins, 1140 were common proteins detected in ocular sarcoidosis, VRL, and controls (Figure 2A). Sixteen proteins were only detected in patients with ocular sarcoidosis, whereas 151 proteins were only detected in VRL. Next, to identify DEPs in ocular sarcoidosis, proteins with an FDR-adjusted *p* value less than 0.05 were extracted as proteins with differential expression. Eventually, 290 and 174 proteins were detected as DEPs in ocular sarcoidosis compared with controls and VRL, respectively (Figure 2B,C, and listed in Table S3). Principal component analysis visually depicted significant separation of ocular sarcoidosis from controls and VRL (Figure 2D,E). Hierarchical clustering showed that of 290 vitreous DEPs in ocular sarcoidosis compared with controls, 263 proteins were upregulated while 27 were downregulated (Figure 2F). When compared with VRL, 39 proteins were upregulated and 135 were downregulated (Figure 2G). These results suggested that the vitreous protein profile in ocular sarcoidosis was significantly different from those of controls and VRL, although VRL and ocular sarcoidosis manifest similar clinical findings including vitreous opacity.

### 3.2. Functional Interaction Pathway Analysis

**Figure 3:**
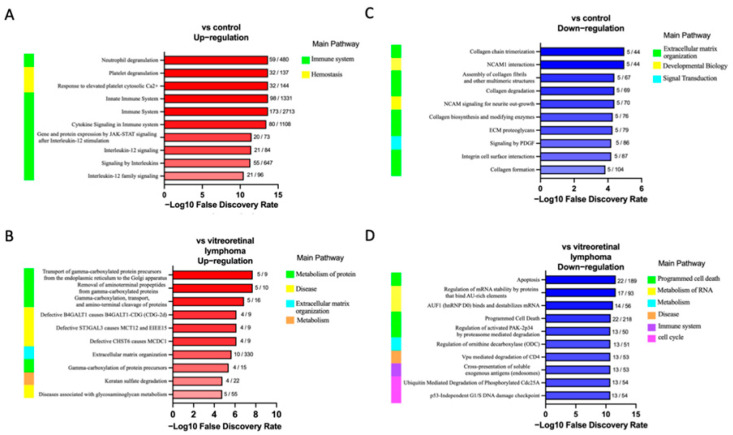
Top 10 altered REACTOME pathways in ocular sarcoidosis identified by enrichment pathway analysis. Biological REACTOME pathways in vitreous samples of ocular sarcoidosis patients were significantly upregulated or downregulated compared with controls (**A**,**C**), and with vitreoretinal lymphoma (**B**,**D**). The main pathway that branches into the detailed pathway is shown on the left of each bar chart. The numbers next to the bars indicate the number of proteins found in that pathway. A gradient associated with the -log10 false discovery rate depicts the red and blue bar colors.

**Figure 4:**
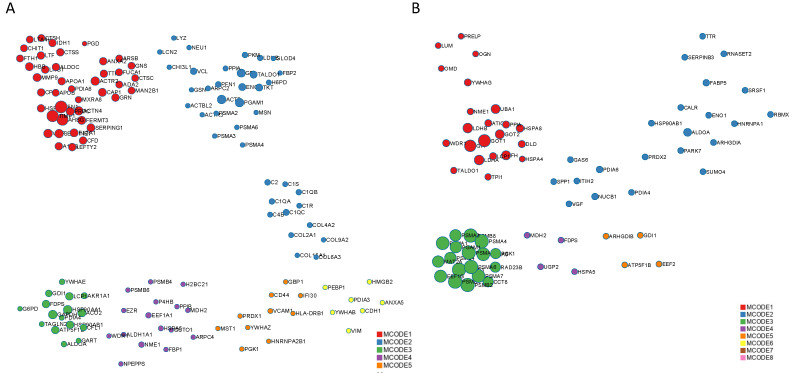
Protein–protein interaction network of differential expression proteins (DEPs) in ocular sarcoidosis. The interactions of these proteins were classified by MCODE and generated by Metascape. MCODE1 of DEPs in ocular sarcoidosis compared with controls included the pathways of “Neutrophil degranulation”, “Platelet degranulation”, and “Response to elevated platelet cytosolic Ca2+” (**A**), whereas MCODE1 of DEPs compared with vitreoretinal lymphoma included the pathways of “Glycolysis and gluconeogenesis”, “Metabolism of carbohydrates”, and “Carbon metabolism” (**B**).

In the second part of our analysis, we aimed to identify the molecular pathways in which each DEP plays a functional role. Detailed analysis of the top 10 pathways associated with the DEPs in ocular sarcoidosis compared with controls and VRL was performed by conducting a search using the Reactome pathway. As shown in Figure 3, among the pathways associated with DEPs in ocular sarcoidosis compared with controls, the “neutrophil granulation” pathway was the most significantly upregulated in ocular sarcoidosis (Figure 3A). Of note, 8 of the top 10 detailed pathways belonged to the main pathway of “Immune system”. In the comparison with VRL, the “Transport of gamma-carboxylated protein precursors from the endoplasmic reticulum to the Golgi apparatus” pathway was the most significantly upregulated pathway in ocular sarcoidosis (Figure 3B). In addition, the top three detailed pathways were included in the main pathway of “Metabolism of proteins”. As for the pathways which were downregulated in ocular sarcoidosis, “Collagen chain trimerization” and “Apoptosis” were the most downregulated pathways compared with controls and VRL, respectively (Figure 3C,D). Protein–protein interaction networks for the DEPs identified in ocular sarcoidosis compared with controls and VRL were visualized using Metascape. The Molecular Complex Detection (MCODE) algorithm was applied to identify densely connected network components. MCODE enrichment analysis based on PPI enrichment analysis for DEPs in ocular sarcoidosis compared with controls showed eight PPI modules (Figure 4A; details listed in Table S4). In particular, the top enrichment term of MCODE1 included 42 proteins distributed in three major clusters of “Neutrophil degranulation”, “Platelet degranulation”, and “Response to elevated platelet cytosolic Ca2+”. When compared with VRL, MCODE enrichment analysis showed five PPI modules (Figure 4B; details listed in Table S4). The top enrichment terms of MCODE1 for DEPs in ocular sarcoidosis compared with VRL included 22 proteins distributed in three major clusters of “Glycolysis and gluconeogenesis”, “Metabolism of carbohydrates”, and “Carbon metabolism”. These results of molecular pathway and PPI network analyses for DEPs in ocular sarcoidosis compared with control and VRL suggest that these DEPs may be deeply involved in the pathogenesis of ocular sarcoidosis.

### 3.3. Validation of the Candidate Biomarker Proteins

**Figure 5:**
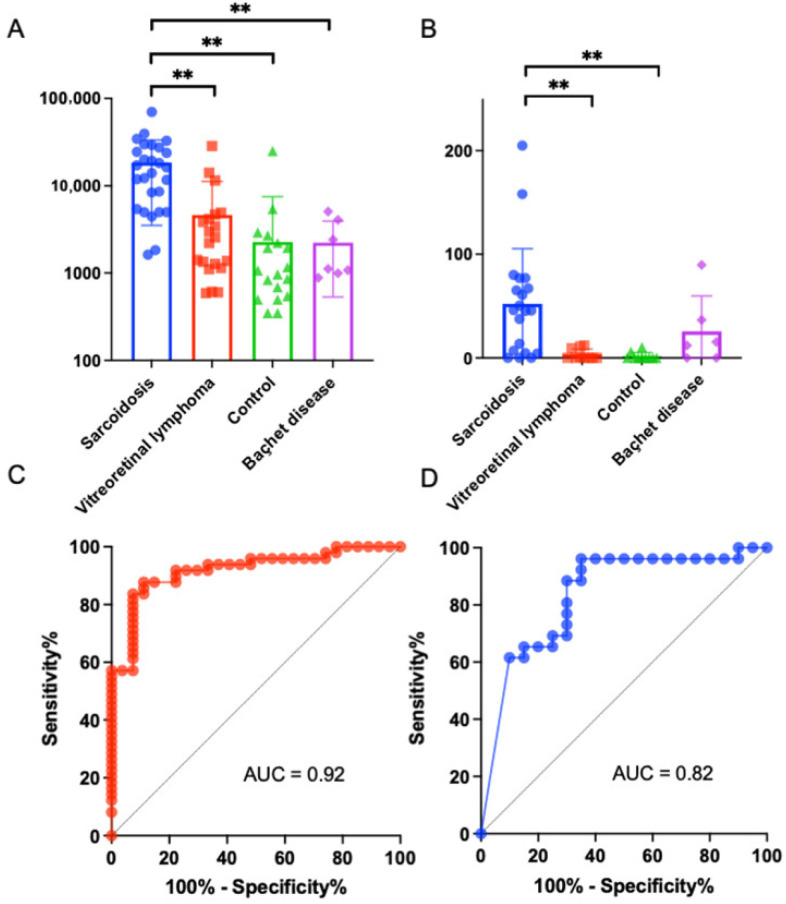
Validation for neutrophil gelatinase-associated lipocalin (NGAL) and junctional adhesion molecule B (JAMB) in vitreous samples as biomarkers for ocular sarcoidosis, using vitreous samples from various diseases. Enzyme-linked immunosorbent assay showed that vitreous NGAL concentration in sarcoidosis increased significantly compared to vitreoretinal lymphoma, controls, and Behçet’s disease (**A**), and vitreous JAMB concentration in ocular sarcoidosis increased significantly compared to vitreoretinal lymphoma and controls, but no significant difference compared to Behçet’s disease (** *p* < 0.01) (**B**). The receiver operating characteristic (ROC) curves of NGAL (**C**) and JAMB (**D**) showed large areas under the ROC curves (AUC) specific to ocular sarcoidosis.

Finally, we aimed to identify the candidate protein biomarkers for distinguishing ocular sarcoidosis from other diseases resembling ocular sarcoidosis. The top 10 DEPs in vitreous that showed the most significant differences (FDR-adjusted *p* value) between ocular sarcoidosis and controls or VRL are listed in Table 2. Neutrophil gelatinase-associated lipocalin (NGAL) and junctional adhesion molecule (JAM) B were selected as candidate biomarker proteins because they had *p* values less than 0.01 indicating significant differences when compared with both controls and VRL. Next, to quantify the vitreous expression levels of these two candidate proteins, ELISA assay was performed in a patient cohort much expanded from the cohort used in high-throughput proteomic analysis. The validation population comprised 27 patients with ocular sarcoidosis, 20 with VRL, 22 controls, and seven with BD. Since a larger volume of vitreous is required for measuring JAMB due to the small amounts of protein in some samples, the number of patients recruited for JAMB was lower compared to NGAL. The NGAL protein concentrations were 18,402 ± 14,612, 4610 ± 6459, 2266 ± 5104 and 2229 ± 1572 pg/mL in ocular sarcoidosis (*n* = 27), VRL (*n* = 20), controls (*n* = 22), and Behçet’s disease (*n* = 7), respectively (Figure 5A). The NGAL protein expression in ocular sarcoidosis was significantly upregulated compared to VRL, controls, and Behçet’s disease. On the other hand, the JAMB protein concentrations were 52.2 ± 51.8, 3.3 ± 5.0, 1.6 ± 3.4 and 25.6 ± 31.1 pg/mL in ocular sarcoidosis (*n* = 20), VRL (*n* = 10), controls (*n* = 10), and Behçet’s disease (*n* = 6), respectively, showing significant upregulation in ocular sarcoidosis compared to VRL and controls (Figure 5B). Areas under the ROC curves of NGAL and JAMB for ocular sarcoidosis comparing with other diseases were 0.92 (95% CI; 0.86–0.98, *p* < 0.0001) (Figure 5C) and 0.83 (95% CI; 0.71–0.96, *p* = 0.0001), respectively (Figure 5D). These results suggested that NGAL and JAMB proteins may be candidate vitreous biomarkers for ocular sarcoidosis. In addition, statistical analysis was performed to examine sex difference in these biomarkers. The NGAL and JAMB protein concentrations in ocular sarcoidosis showed no significant differences between males and females (NGAL; *p* = 0.75, JAMB; *p* = 0.90; detailed data shown in Table S2).

## 4. Discussion

Rapid and accurate diagnosis is essential for ocular sarcoidosis because the treatment is fundamentally different from other types of uveitis including Behçet’s disease and VRL [25]. However, nonspecific findings of uveitis sometimes lead to misdiagnosis. Although several proteomics studies using vitreous humor have been conducted aiming to differentiate autoimmune or noninfectious uveitis [15,17], there are no reports of comprehensive proteomics of vitreous humor focusing on ocular sarcoidosis. In addition, advanced mass spectrometry allows high-throughput analysis, providing data of a large number of proteins to facilitate comprehensive proteomic analysis of different diseases. This article is the first report of a comprehensive high-throughput proteomic analysis of vitreous humor in patients with ocular sarcoidosis compared with disease controls including vitreoretinal diseases and VRL.

Many studies have used comprehensive analysis to identify serum biomarkers for the differentiation of uveitis [26,27,28,29]. Some serum protein biomarkers including angiotensin-converting enzyme and soluble interleukin-2 receptor have been reported to be useful for the diagnosis of sarcoidosis in patients with uveitis [30,31,32]. However, although serum biomarkers have been reported for the discrimination of rare types of uveitis, few biomarkers are used in routine clinical practice [33,34]. One of the reasons for this is that serum markers do not entirely reflect the local pathophysiology of ocular inflammation, due to the retina–blood barrier [13]. Therefore, proteomic analyses of vitreous specimens are preferred to elucidate the differences between different uveitis diseases and to identify biomarkers for each disease.

Our study detected 290 significant DEPs in ocular sarcoidosis compared with controls, most of which were upregulated. Notably, pathway enrichment analysis of the upregulated pathways in ocular sarcoidosis showed that most of the top 10 pathways involved the immune system. In particular, “Neutrophil degranulation”, the most significantly upregulated pathway in ocular sarcoidosis, is associated with the innate immune system. Sarcoidosis is a complex disease involving both the innate and adaptive immune systems [35]. There are numerous reports on the pathogenesis of pulmonary sarcoidosis based on proteomic analysis of bronchoalveolar lavage (BAL) [36,37,38,39], which confirm the involvement of immune systems. On the other hand, C. acnes (previously classified as Propionibacterium acnes) has been reported to be related to the pathogenesis of ocular sarcoidosis [18,40]. However, there are few reports on the molecular pathogenesis of ocular sarcoidosis due to the challenging nature of collecting ocular samples compared to BAL in pulmonary sarcoidosis. Our finding that the upregulated vitreous DEPs in ocular sarcoidosis involve immune pathways suggests that the immune pathways also play an important role in the development of ocular sarcoidosis, as observed in pulmonary sarcoidosis.

In this study, the expression levels of NGAL and JAMB in vitreous humor were confirmed using ELISA. NGAL, also known as lipocalin-2, is an adipokine associated with various processes including metabolic homeostasis, apoptosis, infection, immune response, and inflammation [41,42,43,44]. A report showed increased NGAL expression in response to C. acnes [45]. In particular, NGAL has been reported to be elevated in various inflammatory diseases, suggesting that it may be a potential biomarker [46,47,48,49,50,51]. Batsos et al. [51] reported that NGAL was highly expressed in the vitreous humor of rhegmatogenous retinal detachment. Another report revealed a positive correlation between NGAL concentration and proliferative diabetic retinopathy (PDR) grade, suggesting that NGAL has a potential role in the pathogenesis and progression of PDR48. In that report, the average NGAL concentration in the vitreous of PDR patients was 63,522 pg/mL, which was higher than the vitreous concentration of NGAL in ocular sarcoidosis in our study. However, when NGAL is used as a biomarker for ocular sarcoidosis in the clinical setting, differentiation between PDR from ocular sarcoidosis would be rarely needed because the clinical findings of the two diseases are completely different. A study that compared the vitreous concentrations of inflammatory cytokines between PDR and ocular sarcoidosis reported that IL-4, IL-17A, IL-31, and TNFα were significantly elevated in PDR, indicating the involvement of Th2 and Th17-related immune responses in the pathogenesis of PDR [52]. On the other hand, Th1 immune response was suggested to play a critical role in sarcoidosis, based on the elevation of IFN-γ and sCD40L. Although the detailed difference in pathogenetic mechanism between ocular sarcoidosis and PDR remains unknown, our results together with previous findings imply that ocular sarcoidosis and PDR may involve a common inflammatory pathway not observed in VRL or Behçet’s disease. The current study revealed elevated NGAL protein expression in the vitreous humor of patients with ocular sarcoidosis, suggesting that NGAL is not only a valuable ocular biomarker for sarcoidosis but may also contribute to elucidate the pathogenesis of sarcoidosis.

In addition, JAMB, which is a member of the junctional adhesion molecule of the immunoglobulin superfamily, is mainly expressed in vascular endothelial cells and plays a role in supporting leukocyte recruitment to the extravascular space [53]. Liang et al. [54] reported that JAMB expression was more extensive in tissues with chronic inflammatory diseases including asthma and autoimmune hepatitis. Kim et al. [55] reported that the expression of JAMB in ocular tissue was associated with retinal ganglion cells. However, to the best of our knowledge, there are no reports on the relationship between the pathogenesis of inflammation and JAMB in ocular tissue. Therefore, our results indicate that JAMB in vitreous humor may be a candidate biomarker for ocular sarcoidosis. In addition, given that NGAL in vitreous humor is also elevated in PDR, JAMB and NGAL should be evaluated together as potential diagnostic biomarkers.

Pathways related to the downregulated miRNAs in biopsied specimens of patients with orbital MALT lymphoma compared to IgG4-ROD were mostly related to the MAPK signaling pathway. This signaling is a well-investigated pathway involved in inflammation due to a pathway regulated by the translocation gene of MALT lymphoma.

The analysis of pathways enriched in the target genes in the significantly expressed miRNAs confirmed the essential role of these transcripts and their relative targeting miRNAs in the pathogenesis of IgG4-ROD and orbital MALT lymphoma.

While this study has revealed several interesting findings, the single-institute setting and retrospective design are the main limitations of this study. In addition, although the subjects with ocular sarcoidosis or VRL were not treated with corticosteroids within one month before vitrectomy, as per the selection criteria, the vast majority of the subjects had undergone steroid treatments including eyedrops, sub-Tenon’s injection and oral medications for vitreous opacity prior to the one-month pre-vitrectomy period. These treatments could have affected the vitreous samples. A study on treatment naïve eyes would be ideal. However, analysis of vitreous humor samples from patients who had a recent history of corticosteroid treatment as in this study is commonly encountered in actual clinical practice, because eyes with acute inflammation such as ocular sarcoidosis usually have received prior steroid treatments before undergoing vitrectomy. Further study that excludes patients with inactive ocular sarcoidosis and analyzes the changes in proteomics over the course of treatment for ocular sarcoidosis may promote understanding of the pathogenesis and identification of diagnostic biomarkers. Furthermore, there was sex bias in the patient groups in proteomic analysis. While there were equal numbers of males and females (*n* = 5 each) in the control group, 80% of patients with ocular sarcoidosis were females. A reason for the high percentage of female patients with ocular sarcoidosis is that the disease tends to show female preponderance. In addition, vitrectomy is not frequently performed in ocular sarcoidosis due to the invasive nature of the procedure. For these reasons, adjustment for sex difference in the proteomic specimens was difficult. However, the vitreous concentrations of NGAL and JAMB measured by ELISA were not significantly different between males and females (NGAL; *p* = 0.75, JAMB; *p* = 0.90). Hence, whether sex difference exists in these biomarkers remains unknown. We plan to further validate the present findings with a larger number of cases in future study.

## 5. Conclusions

We report for the first time a comprehensive proteomic analysis of vitreous humor in patients with ocular sarcoidosis compared with VRL and other vitreoretinal diseases. Identification of the DEPs contributes to our understanding of the pathogenesis of ocular sarcoidosis relative to other vitreoretinal inflammatory diseases. In particular, NGAL and JAMB expressions were significantly upregulated in ocular sarcoidosis compared to VRL and controls. This result suggests that these two proteins may be potential biomarkers for ocular sarcoidosis.

## Figures and Tables

**Table 1:** Demographic data of patients in this study.

**Demographic Data of Patients with Sarcoidosis, Vitreoretinal Lymphoma, and Control Included in Comprehensive Proteomics Analysis**					
		**Sarcoidosis**	**Vitreoretinal lymphoma**	**Control**	
Number of cases		10	10	10	
Age (years)		69.5 ± 10.1	63.4 ± 11.1	62.1 ± 13.3	
Sex	Male	2	2	5	
	Female	8	8	5	
**Demographic data of patients with sarcoidosis, vitreoretinal lymphoma, and control included in validation for ELISA**					
		**Sarcoidosis**	**Vitreoretinal lymphoma**	**Control**	**Beçhet disease**
Number of cases		27	20	22	7
Age (years)		67.0 ± 9.7	64.5 ± 10.3	67.8 ± 9.6	45 ± 12.4
Sex	Male	5	10	7	5
	Female	22	10	15	2

**Table 2:** Top 10 vitreous differential expression proteins in ocular sarcoidosis compared with control and vitreoretinal lymphoma.

Upregulated Proteins	FDR		Downregulated Proteins	FDR	
	vs. Control	vs. VRL		vs. Control	vs. VRL
Neutrophil gelatinase-associated lipocalin	0.001	0.008	Mammalian ependymin-related protein 1	0.022	0.173
Junctional adhesion molecule B	0.002	<0.001	Tubulin-specific chaperone A	0.026	<0.001
Chitinase-3-like protein 2	0.002	0.013	Transferrin receptor protein 1	0.027	0.061
Complement C1s subcomponent	0.009	0.043	Carbonic anhydrase 14	0.033	0.131
Collagen alpha-3(VI) chain	0.013	0.021	N-acetylglucosamine-6-sulfatase	0.037	0.178
Vitamin K-dependent protein Z	0.025	0.049	Uncharacterized protein KIAA1958	0.047	0.033
Hyaluronan-binding protein 2	0.025	0.039	Putative phospholipase B-like 2	0.049	0.218
Dihydropteridine reductase	0.039	0.026	Semaphorin-7A	0.065	0.067
Fibroblast growth factor-binding protein 2	0.040	0.036	Contactin-associated protein-like 4	0.069	0.032
Beta-tectorin	0.040	<0.001	Nuclear autoantigenic sperm protein	0.076	0.006

## Data Availability

The data presented in this study are available on request from the corresponding author.

## Supplementary Materials

The following are available online at https://www.mdpi.com/article/10.3390/jcm11133606/s1, Table S1: Clinical information of all individual cases analyzed in this study. Table S2: Results of comprehensive protein expression levels in 8 females and 2 males with ocular sarcoidosis. Table S3: List of differentially expressed proteins in patients with sarcoidosis compared with control and with vitreoretinal lymphoma. Table S4: Molecular Complex Detection (MCODE) network for DEP identified in ocular sarcoidosis compared with controls and with vitreoretinal lymphoma.
